# Stimulation of the dorsolateral prefrontal cortex modulates muscle sympathetic nerve activity and blood pressure in humans

**DOI:** 10.1093/texcom/tgac017

**Published:** 2022-04-14

**Authors:** Gianni Sesa-Ashton, Rebecca Wong, Brendan McCarthy, Sudipta Datta, Luke A Henderson, Tye Dawood, Vaughan G Macefield

**Affiliations:** Baker Heart and Diabetes Institute, Human Autonomic Neurophysiology, 75 Commercial Road, Melbourne, VIC 3004, Australia; Baker Heart and Diabetes Institute, Human Autonomic Neurophysiology, 75 Commercial Road, Melbourne, VIC 3004, Australia; Baker Department of Cardiometabolic Health, The University of Melbourne, Parkville, VIC 3010, Australia; Baker Heart and Diabetes Institute, Human Autonomic Neurophysiology, 75 Commercial Road, Melbourne, VIC 3004, Australia; Baker Department of Cardiometabolic Health, The University of Melbourne, Parkville, VIC 3010, Australia; Baker Heart and Diabetes Institute, Human Autonomic Neurophysiology, 75 Commercial Road, Melbourne, VIC 3004, Australia; Baker Department of Cardiometabolic Health, The University of Melbourne, Parkville, VIC 3010, Australia; School of Medical Sciences (Neuroscience), Brain and Mind Centre, The University of Sydney, NSW 2050, Australia; Baker Heart and Diabetes Institute, Human Autonomic Neurophysiology, 75 Commercial Road, Melbourne, VIC 3004, Australia; Baker Department of Cardiometabolic Health, The University of Melbourne, Parkville, VIC 3010, Australia; Baker Heart and Diabetes Institute, Human Autonomic Neurophysiology, 75 Commercial Road, Melbourne, VIC 3004, Australia; Baker Department of Cardiometabolic Health, The University of Melbourne, Parkville, VIC 3010, Australia

**Keywords:** dorsolateral prefrontal cortex, sympathetic nervous system, transcranial electrical stimulation

## Abstract

**Introduction:**

Muscle sympathetic nerve activity (MSNA) controls the diameter of arterioles in skeletalmuscle, contributing importantly to the beat-to-beat regulation of blood pressure (BP). Although brain imaging studies have shown that bursts of MSNA originate in the rostral ventrolateral medulla, other subcortical and cortical structures—including the dorsolateral prefrontal cortex (dlPFC)—contribute.

**Hypothesis:**

We tested the hypothesis that MSNA and BP could be modulated by stimulating the dlPFC.

**Method:**

dlPFC. In 22 individuals MSNA was recorded via microelectrodes inserted into the common peroneal nerve, together with continuous BP, electrocardiographic, and respiration.Stimulation of the right (*n*=22) or left dlPFC (*n*=10) was achieved using transcranial alternating current (tcACS; +2 to −2mA, 0.08 Hz,100 cycles), applied between the nasion and electrodes over the F3 or F4 EEG sites on the scalp.

**Results:**

Sinusoidal stimulation of either dlPFC caused cyclicmodulation of MSNA, BP and heart rate, and a significant increase in BP.

**Conclusion:**

We have shown, for the first time, that tcACS of the dlPFC in awake humans causes partial entrainment of MSNA, heart rate and BP, arguing for an important role of this higher-level cortical area in the control of cardiovascular function.

## Introduction

The beat-to-beat control of blood pressure (BP) depends on detection of BP through the arterial baroreceptors and rapid compensatory changes in cardiac output and total peripheral resistance, via the negative-feedback baroreflex loop. Changes in heart rate and stroke volume are brought about through increases and decreases in cardiac sympathetic and parasympathetic (vagal) drive to the heart, whereas neurally mediated changes in peripheral resistance are brought about exclusively by increases or decreases in sympathetic vasoconstrictor drive to the muscle (and splanchnic) vascular beds. Direct recordings of muscle sympathetic nerve activity (MSNA), via microelectrodes inserted percutaneously into a peripheral nerve of awake humans, coupled with functional magnetic resonance imaging (fMRI), allow us to identify brain regions involved in the generation of MSNA, and hence to identify the sympathetic connectome involved in BP regulation (Macefield and Henderson 2016, 2019). We have used this approach to functionally identify the rostral ventrolateral medulla (RVLM), where the major population of neurons driving MSNA generation are found (Dampney 1994), as well as other brainstem nuclei involved in cardiovascular regulation, including regions responsible for arterial baroreflex control of beat-to-beat BP (Macefield and Henderson 2010, 2019).

Using MSNA-coupled fMRI we have also identified areas above the brainstem, involved in generating resting MSNA, including the dorsomedial and ventromedial hypothalamus (VMH), insula, posterior cingulate cortex, precuneus, and dorsolateral prefrontal cortex (dlPFC; James et al. 2013). That a higher cortical area such as the dlPFC showed strong temporal coupling to spontaneous bursts of MSNA at rest was somewhat surprising, given this region is considered to be primarily responsible for social and executive functioning (Yuan and Raz 2014; Holper et al. 2018; Piva et al. 2019). In addition, the dlPFC appears to be critical for on-going mood regulation, with dlPFC hypoactivity associated with negative emotional judgment and major depressive disorder (Bench et al. 1995; Grimm et al. 2008; Zhong et al. 2011). Interestingly, major depressive disorder is associated with increased heart rate and reduced heart rate variability, and several studies have reported reduced heart rate following dlPFC stimulation (Licht et al. 2008; Brunoni et al. 2013; Makovac et al. 2017). Although there is emerging evidence for a role of the dlPFC in regulating heart rate, it remains unknown what role, if any, it plays in regulating spontaneous sympathetic drive in healthy humans.

The dlPFC is the most common site for application of transcranial direct current stimulation (tcDCS) and transcranial alternating current stimulation (tcACS), which alters membrane excitability but does not directly cause action potential firing (Knotkova et al. 2019). Although tcDCS delivers a weak current at 1–2 mA between an anode and cathode, tcACS typically delivers a sinusoidal current in which the anode and cathode will switch during each half-sine wave (Woods et al. 2016). Both tcDCS and tcACS promote changes in cortical excitability: tcDCS induces a static change in excitability throughout stimulation, either increasing or decreasing excitability according to electrode polarity (Knotkova et al. 2019), whereas tcACS causes oscillations in neuronal excitability (Colzato 2017). Here we sought to determine the role of the dlPFC in modulating spontaneous MSNA and subsequently the modulation of BP in healthy individuals, notwithstanding the fact that this is in the context of a network. Nevertheless, the provision of such evidence would demonstrate that the dlPFC plays a role in the control of BP at rest and, potentially, in disease states associated with altered autonomic function.

## Materials and methods

In total, 20 males and 14 females [age range 21–36 years of age, 25 ± 4 years mean ± standard deviation (SD)] participated in this study. Of these, MSNA was recorded in 22 participants (13 males and 9 females). Participants were asked to refrain from caffeine and nicotine on the morning of the study. The study was approved by the Human Research Ethics Committee of Western Sydney University (HREC approval H11010), endorsed by Governance of the Baker Heart and Diabetes Institute and conformed to the Declaration of Helsinki, with all participants providing written informed consent.

### Recording procedures

Participants lay semi-recumbent in a chair with their backs at 45° and their legs supported horizontally. After locating the course the of the common peroneal nerve at the level of the fibular head, by delivering electrical pulses (2–10 mA; Stimulus Isolator, ADInstruments, Sydney, Australia) through a 2-mm surface probe, a tungsten microelectrode (Frederick Haer and Co, Bowdoin) was inserted into the skin over the right (*n* = 20) or left (*n* = 2) common peroneal nerve; a nearby subdermal electrode with a larger uninsulated tip served as the reference electrode. Weak electrical stimulation delivered through the microelectrode (0.2 ms, 1 Hz, and 0.01–1.0 mA) was used to guide the manipulation of the tip into the nerve; evoked muscle twitches at 0.01–0.02 mA, without radiating paraesthesia, indicated that the tip had penetrated a fascicle and identified the muscle. Neural activity was amplified (gain 2 × 10^4^, bandpass 0.3–5.0 kHz) via an isolated amplifier and headstage (NeuroAmpEX, ADInstruments) and stored on a computer (10-kHz sampling) using a computer-based data acquisition and analysis system (PowerLab 16SP hardware and LabChart for Macintosh, v7.1.2.5 software; ADInstruments). Spontaneous or stretch-evoked activity of muscle spindle afferents, without afferent activity produced by light stroking of the leg or foot, confirmed the identity of the fascicle exclusively innervating muscle and not the skin. As such, we avoided the extensor hallucis longus (EHL) fascicle, given this supplies skin between digits I and II as well as the EHL muscle. The microelectrode was further manipulated until spontaneous bursts of MSNA with a clear cardiac rhythmicity, increasing during a maximal inspiratory apnoea, were encountered. A mean voltage neurogram of the filtered nerve signal was displayed from the root-mean square (RMS) processed signal (200-ms moving average). Electrocardiographic (ECG; 0.3 Hz–1.0 kHz) was recorded with 35-mm Ag–AgCl adhesive hydrogel electrodes (Covidien, Ireland) on the chest and sampled at 2 kHz (0.3–1.0 kHz). Continuous BP was recorded non-invasively by finger pulse plethysmography, sampled at 400 Hz (DC-200 Hz), and calibrated with an integrated sphygmomanometer cuff on the opposite upper arm (NOVA; Finapres Medical System BV, Amsterdam, the Netherlands). Technical problems meant we were without BP measurements for 12 experiments, so we recruited an additional group of 12 participants (7 males and 5 females) specifically to address the effects of tcACS on BP; MSNA was not recorded in this group. Respiration was sampled at 100 Hz (DC-100 Hz) using a respiratory belt transducer (ADInstruments).

### Stimulation procedures

An electroencephalogram (EEG) cap was placed over the head and the standard 10–20 EEG positions F3 and F4, corresponding to the left and right dlPFC respectively, were marked. After removing the cap, 35-mm adhesive hydrogel Ag–AgCl electrodes (Covidien, Ireland) were placed over these sites as well as over the nasion, which served as the anode. Additional conductive cream was placed on the electrode to optimize electrode contact; an elastic headband ensured the F3 and F4 electrodes remained in contact. In 2 participants, we also delivered stimulation between EEG electrode sites C4 and the vertex (Cz) in a separate session. Following a 10-min baseline recording, sinusoidally modulated stimuli (−2 to +2 mA, 0.08 Hz) were delivered to the electrodes via an isolated constant-current stimulator (Linear Stimulus Isolator, World Precision Instruments, FL), controlled by the PowerLab. Participants either received tcACS of the right dlPFC only (*n* = 12) or, for 10 participants, tcACS of the right and then the left dlPFC, or vice versa, delivered in a random order; a 3-min rest period separated the latter 2 stimulation protocols. Each period of stimulation was delivered for 100 cycles; at 0.08 Hz each stimulus set lasted ~ 21 min.

**Fig. 1:**
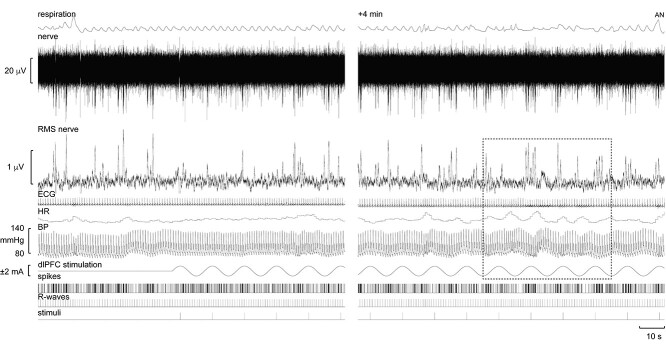
Multi-unit recording of MSNA, together with the RMS-processed neurogram (RMS nerve), ECG, heart rate (HR), blood pressure (BP), and respiration at rest and during tcACS over the ipsilateral dlPFC. Discriminated, negative-going (sympathetic) spikes extracted from the nerve recording are illustrated as standard pulses (spikes). The times of occurrence of each heartbeat (R-waves) and the positive peaks of the sinusoidal dlPFC stimulation at 0.08 Hz are also shown as standard pulses (stimuli). The selection in the right panel, obtained 4 min into the stimulation, emphasizes the cyclic fluctuations in BP and heart rate, and clustering of MSNA, during tcACS over the ipsilateral dlPFC.

### Data analysis

The average number of bursts per minute was computed using the Cyclic Measurements feature of LabChart from the RMS-processed nerve signal. MSNA bursts were included if their peak amplitude exceeded 1.0–1.2 standard deviations above the calculated mean. Burst counts were determined for 3-min periods at baseline prior to any stimulation, the first 3 min of stimulation (Early Phase), the final 3 min of stimulation (Late Phase), and 3 min immediately following the stimulation. These counts were then converted to burst frequency (bursts/minute). To investigate changes in mean BP, the BP trace was also analyzed using the Cyclic Measurements feature. From this data, a modulation index was generated from the greatest cyclical increase in mean BP during stimulation period, compared with the maximal BP excursion at rest. A 3-min section prior to stimulation with stable resting breathing was selected for comparison.

As described previously (Hammam et al. 2011), negative-going spikes in the nerve recording (with a half-width of 0.2–0.5 ms) and the positive peaks of the sinusoidal stimulus or the R-waves of the ECG were detected using window discriminator software (Spike Histogram for Macintosh v2.2, ADInstruments, Sydney, Australia). This was used to generate cross-correlation histograms between MSNA and the ECG; the same discriminator settings used to detect MSNA were then used to construct cross-correlograms between the MSNA and the dlPFC stimulation. These histograms, together with the autocorrelation histograms for the reference signals, were then exported to a statistical and graphical analysis program (Prism 7.0 for Macintosh, GraphPad Software, United States) and the data fitted to smoothed polynomials. Quantification of cardiac modulation of MSNA, or of dlPFC-induced modulation, was performed by measuring the difference in the number of spikes on the smoothed curve at the peak of the modulation and at the trough, which were then expressed as the modulation index (%) = [(peak—trough)/peak] × 100.

All statistical analyses were performed using Prism 9.0 for Macintosh (GraphPad Software). Data were tested for normality using D’Agostino and Pearson Normality tests. An unpaired Mann–Whitney test was performed on the modulation indices of individuals who received either left dlPFC stimulation or right dlPFC stimulation. A paired Wilcoxon test was used to analyze data from individuals who received both left and right tcACS. Comparisons of average MSNA and heart rate between stimulation paradigms were achieved through a mixed effect analysis on unpaired data utilizing Sidak’s multiple comparisons. Differences in BP between baseline and stimulation paradigms were assessed with a paired *t*-test. Data are presented as mean ± SD or as median and percentiles.

## Results

Successful recordings of MSNA were obtained in 22 participants, with the majority (*n* = 20) being obtained from muscle fascicles of the right common peroneal nerve. All participants received 100 cycles of tcACS over the right dlPFC, with 10 of those also receiving tcACS over the left dlPFC. Although participants reported some initial tingling under the stimulation electrodes, which was not painful, this abated within a couple of minutes of stimulation. Other than this, participants reported nothing during the ~ 21 min of stimulation.

**Fig. 2:**
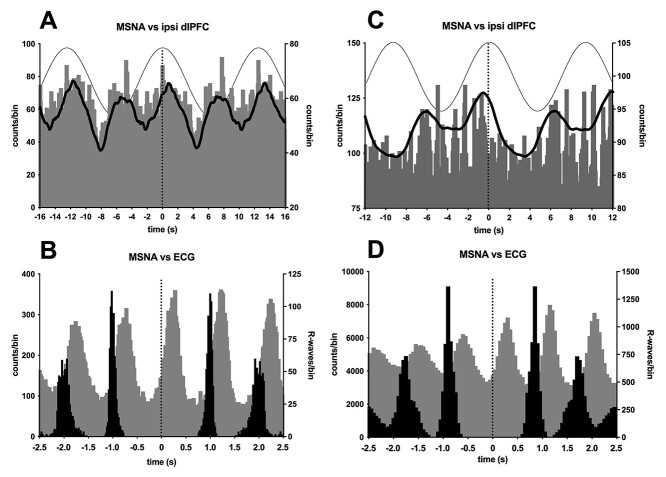
**A**, **C**) Cross-correlation histogram between MSNA and stimulation over the right dlPFC (ipsilateral) at 0.08 Hz in 2 participants. The continuous line shows the smoothed polynomial (counts/bin indicated on right axis). Three cycles of the sinusoidal stimulus are superimposed to illustrate the timing of the stimulation. **B**, **D**) Corresponding cross-correlation histograms between MSNA and ECG (gray bars), with the autocorrelogram of the R-waves shown in black (right axis). Five cardiac cycles are shown. Note that the timescales of the upper and lower panels are different. In all panels time 0 represents the reference event for the cross-correlation histograms (sinusoidal stimulus or R-wave) or auto-correlation histograms (R-wave).

**Fig. 3:**
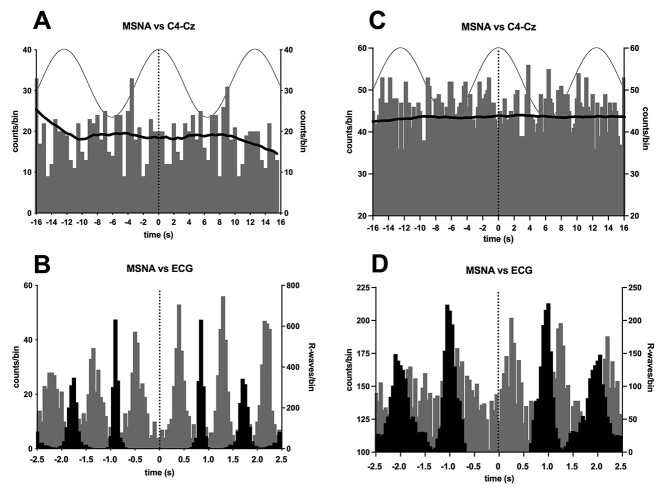
**A**, **C**) Cross-correlation histogram between MSNA and stimulation between the right C4 (hand area) electrode and Cz (vertex) at 0.08 Hz in 2 participants. The continuous line shows the smoothed polynomial (counts/bin indicated on right axis). Three cycles of the sinusoidal stimulus are superimposed to illustrate the timing of the stimulation. **B**, **D**) Corresponding cross-correlation histograms between MSNA and ECG (gray bars), with the autocorrelogram of the R-waves shown in black (right axis). Five cardiac cycles are shown. Note that the timescales of the upper and lower panels are different. In all panels time 0 represents the reference event for the cross-correlation histograms (sinusoidal stimulus or R-wave) or auto-correlation histograms (R-wave).

Experimental records from one participant are shown in Fig. 1, with spontaneous bursts recorded at baseline and the start of stimulation over the right (ipsilateral to the nerve recording) dlPFC shown on the left and 4 min into the 21-min period of stimulation being shown on the right. Stimulation of the dlPFC caused partial entrainment of MSNA, with clustering of the bursts being aligned to the sinusoidal stimulus. The sympathetic spikes extracted from the neurogram are shown as standard pulses at the bottom of the figure, together with pulses corresponding to the R-waves of the ECG and the positive peaks of the dlPFC stimulation. These timing events were used to construct the cross-correlation histograms between MSNA and the dlPFC stimulation in Figs 2 and 3 and the ECG in Fig. 2.

Cross-correlation analysis of the data from this participant confirmed a cyclical modulation of MSNA during stimulation of the right dlPFC, as shown in Fig. 2A. For each cycle of the sinusoidal stimulus, there are 2 peaks of modulation: a primary peak associated with the positive (anodal) phase of the stimulus and a secondary peak associated with the negative (cathodal) phase. It should be noted that the magnitude of this modulation is much smaller than the cardiac modulation of MSNA, shown in Fig. 2B; MSNA is strongly entrained to the cardiac rhythm via the arterial baroreflex. Not all participants showed robust modulation of MSNA: data from another participant (Fig. 2C) showed very weak modulation of MSNA during sinusoidal stimulation of the ipsilateral dlPFC.

To determine the specificity of the dlPFC site in modulating MSNA, in 2 participants, we also applied 100 cycles of tcACS between EEG electrode positions C4 and Cz, corresponding to the hand area of the primary somatosensory cortex and vertex, respectively—far removed from stimulating electrodes placed over the dlPFC and nasion. As shown in Fig. 3, there was no modulation of MSNA during this control stimulation, whereas dlpFC stimulation did cause modulation in these 2 participants.

**Fig. 4:**
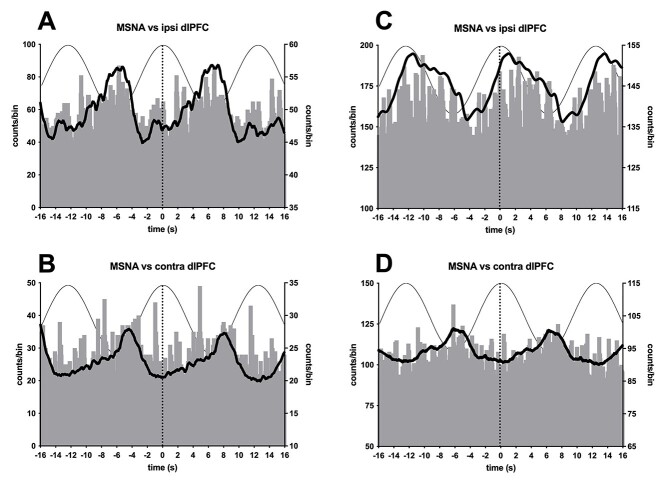
Cross-correlation histograms between MSNA and dlPFC stimulation at 0.08 Hz over the right (ipsilateral) and left (contralateral) dlPFC in 2 participants. The continuous line shows the smoothed polynomial (counts/bin indicated on right axis). Three cycles of the sinusoidal stimulus are superimposed to illustrate the timing of the stimulation.

Stimulation was applied separately to the right (ipsilateral) and left (contralateral) dlPFC in 10 of the 22 participants in whom MSNA was recorded. Cross-correlograms from 2 other participants are shown in Fig. 3. Again, we can see that there is a clear cyclical modulation of MSNA, but unlike the data illustrated in Fig. 2A, in these 2 participants only one peak of modulation was seen for each cycle of stimulation. As noted above, not all individuals exhibited overt modulation. In some, measurable modulation was caused by stimulation over the right dlPFC but not over the left, and vice versa. This is the case in Fig. 4, with both participants showing weaker modulation when tcACS was applied over the left (contralateral) dlPFC. Moreover, for the participant represented in Fig. 2C and D, the peak modulation was aligned to the positive phase of the tcACS when applied to the ipisilateral dlPFC but not to the negative phase when applied contralaterally.

**Fig. 5:**
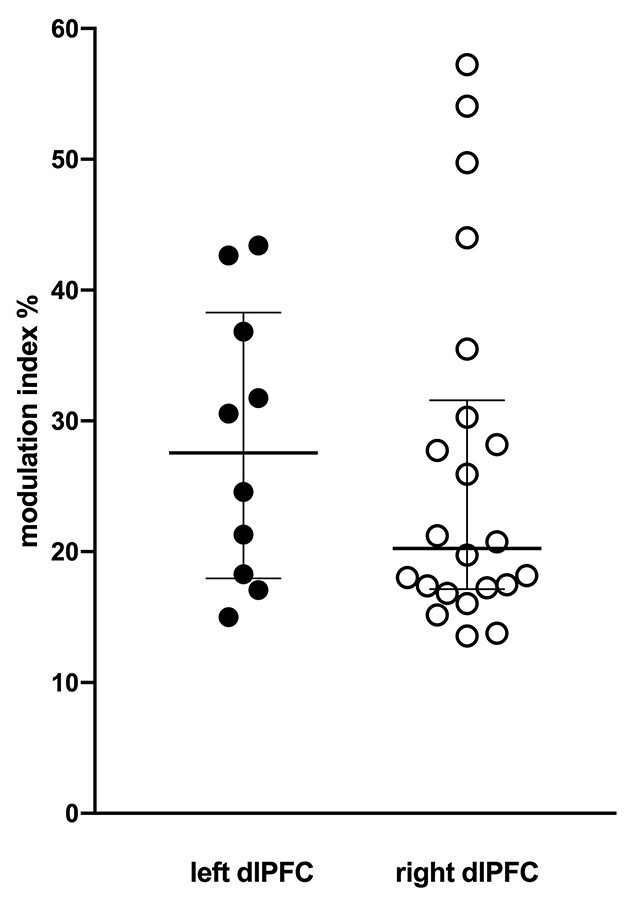
Modulation indices computed for stimulation of the left (*n* = 10) and right (*n* = 22) dlPFC. Central horizontal line represents the median, surrounding box represents the interquartile range.

Despite the side-to-side differences seen in Fig. 4, on average there was no difference in modulation across participants. The median magnitude of modulation produced by tcACS over the right (20.3% [confidence intervals 17.2%, 31.6%], *n* = 22) and the left (27.6% [18.0%, 38.3%], *n* = 10) dlPFC was not significantly different (*P* = 0.411; Mann–Whitney). The same was true for the paired analysis (*P* = 0.557; Wilcoxon, *n* = 10). These data are presented graphically in Fig. 5. Interestingly, the modulation produced by stimulation over the right dlPFC was more variable: while the majority of participants were clustered around a modulation index of ~20%, some had modulation indices > 40%, with the highest being 57%. Conversely, the modulation indices produced by stimulation over the left dlPFC was more limited, ranging from 15 to 43%. There was no significant change in the magnitude of cardiac modulation during dlPFC stimulation (*P* = 0.3373).

Overall MSNA, measured as burst frequency, did not significantly increase above baseline (median [95%CI]: 33.8 [30.6, 76.0] bursts/min) during the first 3 (36.0 [34.0, 80.0]) or last 3 (35.3 [29.0, 81.0]) minutes of stimulation over the right dlPFC; the same was true for stimulation over the left dlPFC (mean ± SD: 32.2 ± 5.0, 34.0 ± 3.4, and 34.4 ± 5.3 bursts/min).

**Fig. 6:**
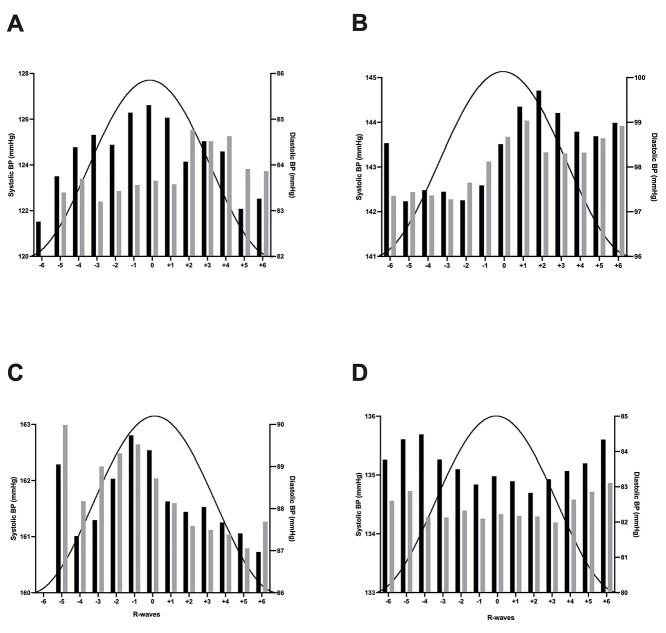
Cross-correlation histograms between systolic BP (black bars) or diastolic BP (gray bars) and the R-waves in 4 participants **A**–**D**) during sinusoidal stimulation of the right dlPFC. The positive phase of the sinusoidal stimulation cycle is superimposed to illustrate the timing of the stimulation. Data are not available for −6 s in **C**) because this participant had a very low heart rate.

In addition to cyclic modulation of MSNA during dlPFC stimulation, it is apparent from Fig. 1 that cyclical changes in BP and heart rate were also observed. Importantly, this cyclic modulation is independent of respiratory-induced fluctuations in BP. Interestingly, across individuals, the BP peak within a stimulation cycle occurred at a different number of R-waves away from the stimulus peak. Although some showed BP responses coinciding with the positive phase of the stimulus peak, others displayed peaks ~2 R-waves before or after the peak. Moreover, 5 participants showed a split response, in which their BP peaked ~5 R-waves either side of the stimulation peak. Examples of the diverse cyclic modulation patterns are provided in Fig. 6.

**Fig. 7:**
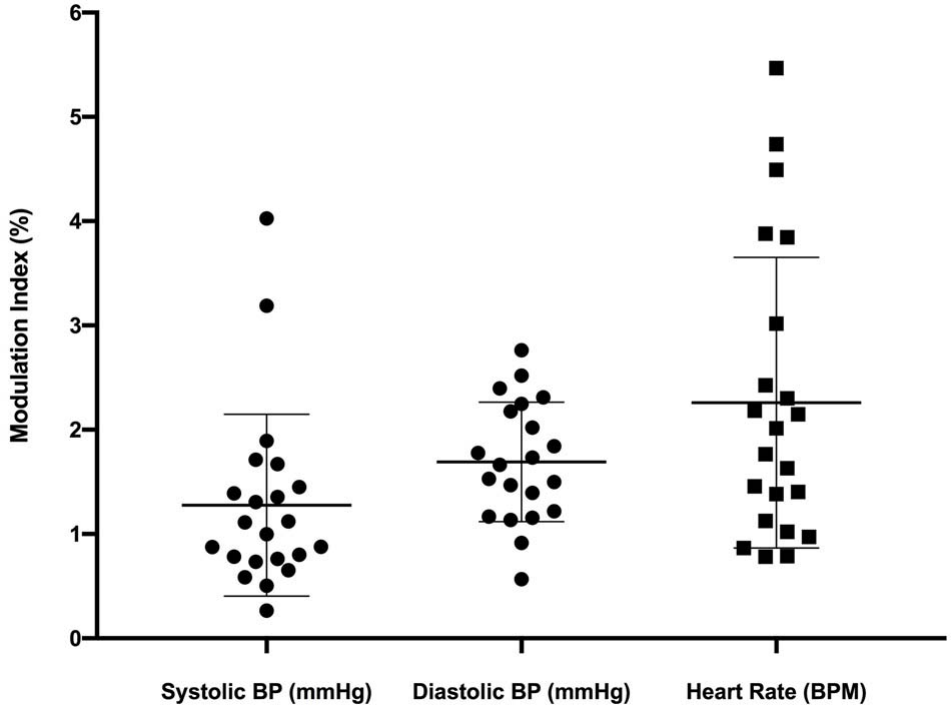
Modulation Indices (mean ± SD) for systolic and diastolic BP as well as heart rate across all 100 cycles of dlPFC stimulation (*n* = 21). *P* < 0.0001 for all variables, derived by one-sample *t*-tests.

Given this variability in timing across participants, the pooled modulation of BP was modest: the mean (± SD) modulation indices were 1.3 ± 0.9% and 1.7 ± 0.6% for systolic and diastolic BP, respectively, which was significantly greater than zero (*P* < 0.0001; *n* = 21, one-sample *t*-test). The same was true for heart rate (2.3 ± 1.4%, *P* < 0.0001; *n* = 21, one-sample *t*-test). Mean data are shown graphically in Fig. 7.

**Fig. 8:**
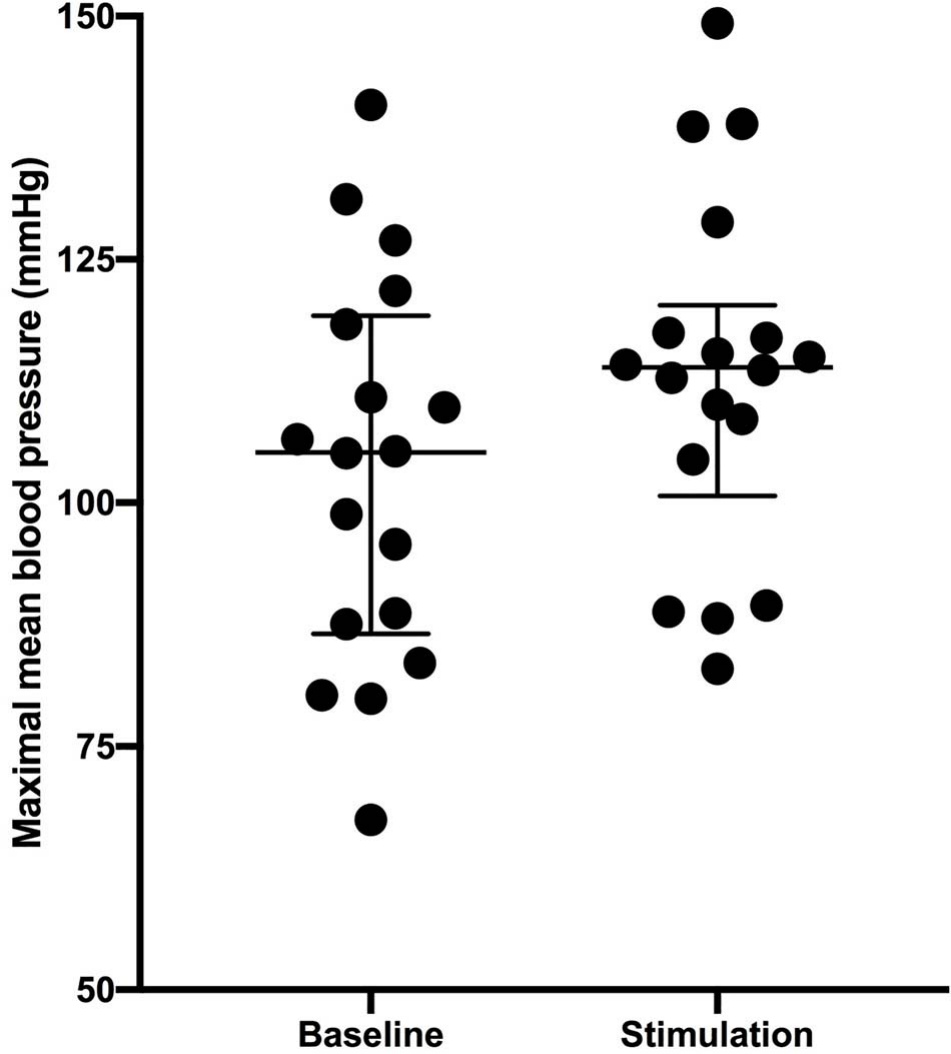
Maximal fluctuations in mean BP at baseline and during stimulation of the right dlPFC. Median ± interquartile range (*n* = 21).

When the maximal fluctuation in mean BP was calculated during the 10 min of baseline and during the ~ 21 min of stimulation of the right dlPFC, it was clear that stimulation caused a significant increase in maximal BP (*P* = 0.0237; paired Wilcoxon test). This is shown in Fig. 8. The median value of mean BP was 105 mmHg at baseline and 114 mmHg during stimulation, an increase of 9 mmHg.

## Discussion

We have shown, for the first time, that tcACS of the dlPFC in awake humans causes partial entrainment of MSNA, heart rate and BP. These changes are independent of respiration, which was not influenced by the stimulation. The magnitude of dlPFC modulation of MSNA (~21%), whereas smaller than the cardiac (~90%) and respiratory (~50%) modulation of MSNA (Fatouleh and Macefield 2013), was similar to that produced by sinusoidal galvanic vestibular stimulation using the same frequency (0.08 Hz), current (±2 mA), and number of cycles (100) as in the current study (~25%; Hammam et al. 2011). The results presented here extend our previous work, which suggested that the dlPFC is part of the sympathetic connectome involved in BP regulation, through its strong coupling to MSNA (James et al. 2013; Macefield and Henderson 2019). Here we show that stimulation of the dlPFC can have a significant effect on MSNA, heart rate and BP and further expands the potential role of this region in health and disease.

### Effects of electrical stimulation of dlPFC

Studies utilizing tcDCS over the left dlPFC have indicated significant falls in resting heart rate during stimulation (Carnevali et al. 2019) and attenuated sympathetic responses, as measured by salivary cortisol, to negative visual stimuli (Brunoni et al. 2013). These analyses, however, have focused on effector responses and utilized direct current stimulation, causing a sustained change in resting membrane potential of cortical neurons and making it impossible to determine oscillatory effects of dlPFC stimulation on MSNA. The use of tcACS is notably less widespread than tcDCS and has been used in a variety of complex cognitive contexts involving the dlPFC, such as risk-taking (Sela et al. 2012), conflict processing (Lehr et al. 2019), and multitasking performance (Hsu et al. 2017). Clinically, tcACS stimulation over the dlPFC appears to have a potential therapeutic role in major depressive disorder (MDD), appearing to rectify changes in alpha oscillations (Alexander et al. 2019). Furthermore, MDD is characterized by hypoactivation of the left dlPFC (Zhong et al. 2011) and returning this activity to physiological levels has been a therapeutic target and has been partially rectified following tcDCS (Holtzheimer and McDonald 2014). There is also growing evidence that infra-slow oscillatory activity (<0.1 Hz), similar to the stimulation frequency employed in this investigation, is a fundamental property of cerebral function (Lőrincz et al. 2009). Indeed, it has been proposed that in some disease states, altered activity is driven by changes in infra-slow oscillatory astrocyte gliotransmission (Parri et al. 2001; Halassa et al. 2007). It is possible that our stimulation paradigm mimics the altered infra-slow oscillatory activity seen in some disease states, which themselves are characterized by perturbed autonomic function.

### Potential pathways by which dlPFC could influence MSNA

We now know that the lesion experiments performed in the past to understand the operation of the brain cannot really quantify the relative contribution of a particular area to the functioning of an entire network, given the extensive interconnections involved. Of course, we are not conducting lesions studies here, but rather attempting to modify the operation of the network electrically by changing the contribution of one area—the dlPFC—to the network. As noted above, we have used this same approach to interrogate the operation of the vestibular system, applying sinusoidal galvanic vestibular stimulation and noting that this causes partial entrainment of MSNA. Indeed, the current project is simply an extension of this series of studies: we are using the same stimulus intensity and frequency of stimulation simply to interrogate the contribution of the dlPFC. We do not think one could have predicted that we would see the effects we have, but ours was a classic hypothesis-driven study informed by our previous research.

Investigating the roles of higher brain regions in anesthetized experimental animals poses significant challenges, particularly those related to anatomical homology between species and the effects of anesthesia on brain function. To overcome these potential limitations, we previously used MSNA-coupled fMRI to reveal several discrete, yet widespread cortical and subcortical areas, in which activity is coupled with spontaneous MSNA bursts. In addition to the left and right dlPFC, these MSNA-activity-coupled regions include the left insula, posterior cingulate cortex, precuneus, left dorsomedial hypothalamus (DMH), left and right VMH, as well as in the RVLM, caudal ventrolateral medulla (CVLM), and nucleus tractus solitarius (NTS; Macefield and Henderson 2010, 2016, 2019; James et al. 2013). It is well established that the RVLM is the final output nucleus for sympathetic vasoconstrictor drive throughout the body, including that to muscle (Dampney 1994; Dampney et al. 2003a, 2003b; Dampney and Horiuchi, 2003), and that the NTS–CVLM–RVLM serial pathway subserves the baroreflex control of sympathetic outflow. Importantly, the cortical regions we had identified, including the dlPFC showed strong functional coupling with the hypothalamus and RVLM (Macefield and Henderson 2016). Although a meta-analysis of human neuroimaging studies has demonstrated that the dlPFC, amygdala, insula, and mid-cingulate cortex are key players in sympathetic control (Beissner et al. 2013), we were the first to show regional cortical signal fluctuations-coupled resting sympathetic outflow in the awake state.

Given that the dlPFC does not project directly to the RVLM, its effects on MSNA must be indirect, potentially via the hypothalamus. Tract tracing studies have revealed that the DMH, which also displays MSNA-coupled fMRI signal intensity changes, receives inputs primarily from the septal area, prefrontal cortex, and subiculum (Horst and Luiten 1986; Thompson et al. 1996; Thompson and Swanson 1998), a finding supported by a recent human diffusion tensor imaging investigation (Lemaire et al. 2011). In addition, although DMH descending projections are relatively sparse, they do contact regions involved in autonomic regulation including the midbrain periaqueductal gray matter (PAG), dorsolateral pons, NTS, RVLM, and CVLM (Hosoya and Matsushita 1981; ter Horst et al. 1984; Hosoya 1985; ter Horst and Luiten 1986; Bandler et al. 2000; de Git et al. 2018). Consistent with these projection patterns, we recently reported significant resting functional connectivity between both the dorsomedial and ventromedial hypothalamus with the dlPFC, RVLM, and insula (James et al. 2013; Macefield and Henderson 2019). It is possible that the effects of oscillatory stimulation of the dlPFC on MSNA rely on a connection to the RVLM via the hypothalamus.

Alternatively, it is possible that dlPFC effects on MSNA are mediated through the hypothalamus via connections to the insula. Although the involvement of the insula in cardiovascular control is well recognized, like the dlPFC, the insula does not send direct projections to the RVLM or directly to sympathetic neurons in the spinal cord (Saper 1982). However, studies in the rat and the monkey have reported an indirect pathway to the RVLM from the insula via the hypothalamus (Cechetto & Chen, 1990; Öngür et al. 1998). Indeed, the anterior insula projects to multiple sites within the hypothalamus, including the ventromedial, lateral, dorsomedial, and posterior hypothalamic regions (Öngür et al. 1998). Consistent with these connections, in a recent resting state functional connectivity study using the RVLM as the “seed” we reported significant connectivity with the dorsomedial and ventromedial hypothalamus, PAG, and the left anterior insula (Macefield and Henderson 2019). Moreover, although we also found connectivity between the RVLM and precuneus, as well as between the hypothalamus and precuneus, a study in the monkey showed that the precuneus neither receives inputs nor projects to the insular cortex, PAG, medulla or spinal cord and there is only a sparse projection from the lateral hypothalamus to the precuneus (Leichnetz 2001). Importantly, the precuneus is strongly coupled to the dlPFC, and both are key elements of the central executive network; the strength of their connectivity is reduced in post-traumatic stress disorder (Olson et al. 2019). Moreover, the precuneus is tightly coupled to the posterior cingulate cortex, which projects directly to the PAG (An et al. 1998).

It is well established that the thresholds for activation of the PAG are regulated by a number of descending sources, including from the hypothalamus (primarily dorsal subregions), as well as parts of the dorsolateral and medial prefrontal cortices (An et al. 1998; Öngür et al. 1998; Floyd et al. 2000). The PAG is known to evoke significant changes in sympathetic outflow and modulate baroreflex sensitivity upon activation, particularly in the context of defensive behaviors (Inui et al. 1994; Keay and Bandler 2001) and part of the hypothalamic regulation of the PAG includes altering cardiovascular control (Ellison and Flyn 1967). The PAG contains 4 longitudinal columns, each column receiving inputs from specific hypothalamic nuclei. The ventromedial hypothalamus projects most strongly to the dorsolateral PAG column and stimulation of this column increases sympathetic drive and mediates behaviors associated with psychological stressors (Dampney 2015, 2018). The DMH projects to the lateral PAG column and stimulation of this column evokes sympathoexcitation and mediates active coping behaviors to escapable physical stressors (Horiuchi et al. 2009). The lateral hypothalamus projects to the ventrolateral PAG column and stimulation of this column causes inhibition of sympathetic outflow and mediates passive coping behaviors to inescapable physical stressors (Keay and Bandler 2001). Electrical stimulation of the human ventrolateral PAG causes a fall in MSNA burst frequency without affecting burst amplitude, whereas stimulation of the dorsolateral PAG increased burst amplitude without affecting burst frequency (Sverrisdóttir et al. 2014). These data support the idea that dlPFC exerts its actions on the RVLM indirectly, likely via the PAG.

### Roles for the dlPFC in disease

Although interindividual differences in dlPFC structure and connectivity are present in healthy populations and are correlated to differences in higher order cognition (Kühn et al. 2014; Turnbull et al. 2019; Zhu et al. 2019), there is evidence of disturbed function in different disease states. Functional connectivity analyses of resting-state fMRI data have revealed differences in the dlPFC in psychiatric disease (Satterthwaite et al. 2015; Misaki et al. 2018), chronic pain sufferers (Hubbard et al. 2014; Ihara et al. 2019), and in substance use disorders (Cisler et al. 2013; Ma et al. 2018). Beyond connectivity, morphological differences are also apparent in pathopsychological states. Notably, a growing body of work indicates that altered anatomy of the dlPFC in individuals with chronic pain (Gustin et al. 2010; Seminowicz and Moayedi 2017), major depressive disorder (Qiu et al. 2014; Reynolds et al. 2014; Zuo et al. 2018), and schizophrenia (Xiao et al. 2013). Moreover, some individuals with major depressive disorder have greatly elevated sympathetic outflow to the heart and other organs, as evidenced from measurement of regional noradrenaline spillover (Barton et al. 2007). We have shown increased MSNA-coupled dlPFC activity in obstructive sleep apnoea (OSA), a condition associated with a marked increase in MSNA and BP (Fatouleh et al. 2014). Moreover, this dlPFC increase is reduced following successful CPAP (continuous positive airway pressure) therapy, which also reduces MSNA towards baseline levels (Fatouleh et al. 2015). It is likely that changes in cortical areas, including the dlPFC, feature in other diseases associated with elevated MSNA and BP.

### Limitations

Although the results show a significant modulation of MSNA, BP and heart rate during stimulation of either the left or right dlPFC, it is difficult to assess how much of this response is exclusive to dlPFC entrainment by tcACS. Most investigators use a bilateral montage to stimulate the dlPFC, with the anode being placed over the dlPFC of one side and the cathode over the opposite dlPFC. We specifically employed unilateral stimulation to allow us to examine potential side-to-side differences; in the end there were none. We used the 10–20 EEG system to locate the F3 and F4 electrode positions, facilitated by using an EEG cap to mark the sites for electrode placement, so we have no doubt that the electrode positions were suitable. For each side, the sinusoidal stimulus was delivered between the dlPFC electrode and an electrode on the nasion: this means that current would essentially spread in an ellipse between the 2 electrodes, and it is highly likely that the current would also affect the ventromedial prefrontal cortex (vmPFC), which could potentially influence our results. However, MSNA-coupled fMRI did not reveal any coupling between MSNA and the vmPFC, at least in healthy individuals (James et al. 2013). It is, however, worth pointing out that there is considerable inter-individual variation in dlPFC size and shape, especially with respect to psychopathologies (Zhong et al. 2011; Zuo et al. 2018). Although EEG-based electrode positioning is robust and consistently used for dlPFC stimulation (Fehér et al. 2017; Alexander et al. 2019), this variation could, in some individuals, dampen the effect of tcACS. Indeed, this may have contributed to the large variation in modulation of MSNA we saw. Finally, all our participants were neurologically normal, healthy normotensive young adults; it remains to be seen whether the modulation of MSNA, BP and heart rate produced by tcACS is augmented in people with hypertension or other disorders.

## Conclusions

By showing that sinusoidal stimulation of the dlPFC can modulate MSNA, this study has revealed an important role for higher executive areas of the brain in cardiovascular control. Given that chronic stress can lead to elevated sympathetic outflow and hypertension (Esler et al. 2008; Esler 2017), our evidence provides the neural substrates by which this can be brought about, likely through the network of connections between the dlPFC and other cortical and subcortical areas that we have previously shown to be involved in the control of MSNA and hence BP. In addition to the cyclic modulation of MSNA, we also found evidence of cyclic modulation of BP and heart rate, and a slight, albeit significant, increase in BP. This suggests that the increase in sympathetic outflow is not limited to the muscle vascular bed: sympathetic outflow to other vascular beds, including the splanchnic circulation, may also be modulated by the stimulus, as may sympathetic drive to the heart. Given that the increases in MSNA often coincided with the increases in heart rate, the most parsimonious explanation is that the tcACS is acting through the sympathetic connectome, of which we know the dlPFC is a part. Of course, this is not to discount the possibility that some (or all) of the effects may be secondary to vagally-mediated effects on the heart, recognizing that vagal control of the heart is also mediated by some of these same areas, but in the absence of direct recordings of vagal activity during stimulation of the dlPFC this remains speculation. Nevertheless, the influence of the dlPFC on resting MSNA, BP and heart rate, shown here, may have significant impacts in disease states, particularly those involving alterations in higher order cognitive and emotional function.

## Data Availability

Data are available on reasonable request.
